# Single-cell transcriptomic profiles in the pathophysiology within the microenvironment of early diabetic kidney disease

**DOI:** 10.1038/s41419-023-05947-1

**Published:** 2023-07-17

**Authors:** Yi-Chun Tsai, Mei-Chuan Kuo, Juan-Chi Huang, Wei-An Chang, Ling-Yu Wu, Yung-Chi Huang, Chao-Yuan Chang, Su-Chu Lee, Ya-Ling Hsu

**Affiliations:** 1 School of Medicine, College of Medicine, Kaohsiung Medical University, Kaohsiung, Taiwan; 2 Division of General Medicine, Kaohsiung Medical University Hospital, Kaohsiung Medical University, Kaohsiung, Taiwan; 3 Division of Nephrology, Kaohsiung Medical University Hospital, Kaohsiung Medical University, Kaohsiung, Taiwan; 4 Center for Liquid Biopsy and Cohort Research, Kaohsiung Medical University, Kaohsiung, Taiwan; 5 Drug Development and Value Creation Research Center, Kaohsiung Medical University, Kaohsiung, Taiwan; 6 Division of Pulmonary and Critical Care Medicine, Kaohsiung Medical University Hospital, Kaohsiung Medical University, Kaohsiung, Taiwan; 7 Graduate Institute of Clinical Medicine, College of Medicine, Kaohsiung Medical University, Kaohsiung, Taiwan; 8 Graduate Institute of Medicine, College of Medicine, Kaohsiung Medical University, Taiwan, Kaohsiung, Taiwan; 9 Department of Anatomy, Kaohsiung Medical University, Kaohsiung, Taiwan

**Keywords:** Transcriptomics, Kidney diseases

## Abstract

Diabetic kidney disease (DKD) is the leading cause of end-stage kidney disease, resulting in a huge socio-economic impact. Kidney is a highly complex organ and the pathogenesis underlying kidney organization involves complex cell-to-cell interaction within the heterogeneous kidney milieu. Advanced single-cell RNA sequencing (scRNA-seq) could reveal the complex architecture and interaction with the microenvironment in early DKD. We used scRNA-seq to investigate early changes in the kidney of db/m mice and db/db mice at the 14th week. Uniform Manifold Approximation and Projection were applied to classify cells into different clusters at a proper resolution. Weighted gene co-expression network analysis was used to identify the key molecules specifically expressed in kidney tubules. Information of cell–cell communication within the kidney was obtained using receptor-ligand pairing resources. In vitro model, human subjects, and co-detection by indexing staining were used to identify the pathophysiologic role of the hub genes in DKD. Among four distinct subsets of the proximal tubule (PT), lower percentages of proliferative PT and PT containing AQP4 expression (PT^AQP4+^) in db/db mice induced impaired cell repair activity and dysfunction of renin-angiotensin system modulation in early DKD. We found that ferroptosis was involved in DKD progression, and ceruloplasmin acted as a central regulator of the induction of ferroptosis in PT^AQP4+^. In addition, lower percentages of thick ascending limbs and collecting ducts with impaired metabolism function were also critical pathogenic features in the kidney of db/db mice. Secreted phosphoprotein 1 (SPP1) mediated pathogenic cross-talk in the tubular microenvironment, as validated by a correlation between urinary SPP1/Cr level and tubular injury. Finally, mesangial cell-derived semaphorin 3C (SEMA3C) further promoted endothelium-mesenchymal transition in glomerular endothelial cells through NRP1 and NRP2, and urinary SEMA3C/Cr level was positively correlated with glomerular injury. These data identified the hub genes involved in pathophysiologic changes within the microenvironment of early DKD.

## Introduction

Diabetes mellitus (DM) is a major healthy burden affecting more than 425 million people worldwide, a number expected to rise to 629 million by 2045 [1]. Diabetic kidney disease (DKD), as one of major microvascular complications of DM, occurs in 30–40% of diabetic patients, and accounts for 30–40% of end-stage kidney disease (ESKD), which globally induces huge socio-economic impacts [2]. Thus, deeper understanding of the pathophysiologic mechanisms of DKD is needed to develop precise therapeutic strategies for DKD.

The kidney is a highly complex organ consisting of around one million nephrons, and the pathogenesis underlying kidney organization, function, and disease involves the complicated cell-to-cell interaction within the heterogeneous kidney milieu [3]. However, the common diagnostic tools for DKD are limited to morphological changes or well-known biomarkers such as serum creatinine and urine albumin, which are usually detected after DKD progression. Advanced single-cell RNA sequencing (scRNA-seq) has been shown to be a powerful tool which can provide information on complex cell-to-cell communication and explore novel biological markers in various diseases [3–5]. Dynamic gene expression in a single cell can correspond to development, differentiation, or disease progression [6]. Accumulating evidence reveals single-cell transcriptomics has been a powerful technology in the field of DKD [7]. Fu et al. and Wilson et al. focused on isolated glomerular cells and identified several intra-glomerular communication signal pathways from induced diabetic eNOS^−/−^ mice and human DKD, respectively [8, 9]. Chung et al. demonstrated the dynamic change of number of glomerular cells in ob/ob mice at different ages [10]. Barwinska et al. established the map of interstitial hub genes involved in extracellular matrix organization and small-molecule catabolism in DKD [11]. Wu et al. further conducted transcriptome study of db/db mice treated with angiotensin receptor blocker (ARB) and sodium-glucose cotransporter two inhibitors (SGLT2i), and indicated that ARB provided more anti-inflammatory and anti-fibrotic effects, whereas SGLT2 affected mitochondrial function in proximal tubules [12]. Another study also used single-cell transcriptomics and found heterogeneity of kidney cell responses to DKD treatment [13]. However, data on cross-talk from scRNA-seq regarding DKD, especially the early stage, have not been fully explored and extra-glomerular cell–cell interactions provide relatively little information regarding the onset of the progression of DKD. Therefore, the aim of this study was to use scRNA-seq to determine the factors and molecular networks involved in the development of early DKD, and further conducted in vitro, in vivo, and human experiments to confirm the pathophysiologic effects of these hub genes on the alteration of microenvironment in DKD.

## Results

### Identification and characterization of cell clusters in the scRNA profile of kidney tissues from db/m and db/db mice

**Fig. 1:**
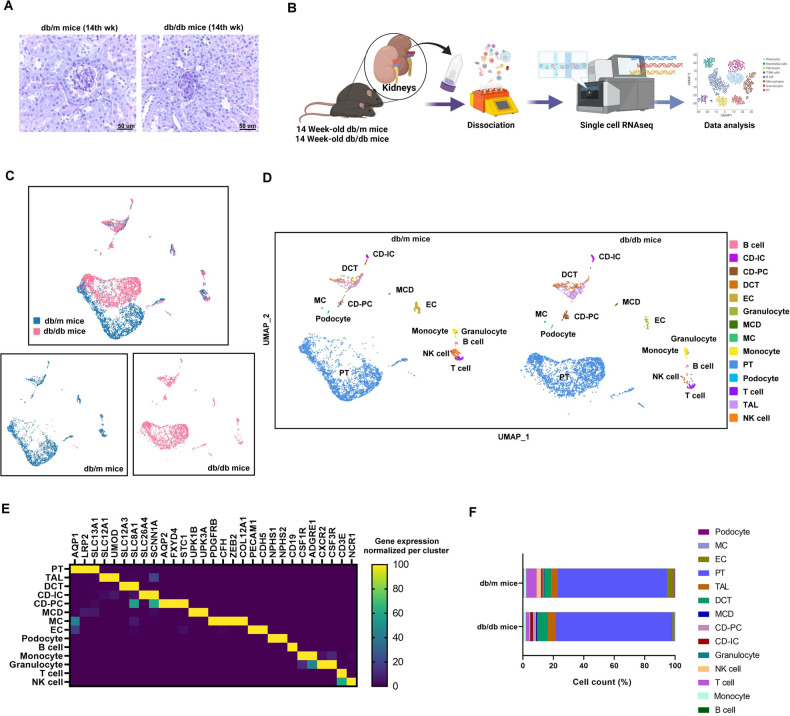
Identification and characterization of cell clusters in the scRNA profile of kidney tissues from db/m mice and db/db mice. **A** Periodic acid–Schiff (PAS) stain of the kidney of db/m mice and db/db mice at 14th week of age. **B** Flowchart of our experiment. **C** Blueprint of the scRNA-sequence of the kidney. **D** Cell clusters of the kidney of db/m mice and db/db mice. UMAP representation of the kidney of db/m mice and db/db mice. **E** Markers of cell cluster. **F** Cell number of each cell cluster.

In order to confirm the early stage of the DKD model, we analyzed db/m mice and db/db mice at different ages. In the 14th week, the db/db mice had higher body weight and fasting glucose than db/m mice (Supplementary Fig. 1A, B), and db/db mice had higher levels of blood urea nitrogen, serum creatinine (Cr) and urinary albumin/Cr ratio (ACR) than db/m mice (Supplementary Fig. 1C–E). We found the stepwise increase in urinary albumin/creatinine ratio (ACR) from db/db mice at 14th week to those at 22th and 33th week (Supplementary Fig. 1F). In Periodic acid–Schiff (PAS) stain, the mesangial matrix expansion was found in db/db mice than db/m mice at the 14th week (Fig. 1A), and the mesangial matrix expansion was more severe in db/db mice at 22th and 33th week than db/db mice at 14th week (Supplementary Fig. 1G). In addition, db/db mice at the 22th and 33th week had severe tubular dilatation and atrophy (Supplementary Fig. 1G). Thus, three kidneys from db/m mice and three from db/db mice at the 14th week were used to establish generate a single-cell transcriptome atlas in the early stage of DKD. The tissue processing pipeline for examining single cell using scRNA-seq is shown in Fig. 1B. The scRNA-seq data were merged, normalized, batch-effect corrected, and clustered to identify the cell types. After quality control filtering, an average of 6633 unique transcripts per cell were detected and found to resolve into 14 distinct cell clusters.

Fourteen separate cell clusters were identified after pooling all of the samples (Fig. 1C, D). Fourteen distinct cell types, including six tubular cell types (5938), three lymphoid cell types (323), two myeloid cell types (130), podocyte (19), mesangial cell (MC, 21), and one endothelial cell (EC) type (202) were annotated based on their known markers. Canonical markers of cell populations were used to identify the major cell types in the kidney (Fig. 1E): proximal tubule (PT; AQP1, LRP2, SLC13A1), thick ascending limb (TAL; SLC12A1, UMOD), distal convoluted tubule (DCT; SLC12A3, SLC8A1), connecting tubule-intercalated cell (CD-IC; SLC26A4, SCNN1A), connecting tubule-principal cell (CD-PC; AQP2, FXYD4, STC1), medullary collecting duct (MCD; UPK1B, UPK3A), MC (PDGFRB, CFH, ZEB2, COL12A1), podocyte (NPHS1, NPHS2), EC (PECAM1, CDH5), B cell (CD19), T cells (CD3E), NK cell (NCR1), monocyte (CSF1R, ADGRE1), and granulocyte (CXCR2, CSF3R). Figure 1F shows the percentages of different cell types in the mice kidneys. Compared to db/m mice, the percentages of glomerular cells including EC, MC and podocyte were decreased in db/db mice; conversely, the percentages of PT, TAL and DCT were increased in db/db mice.

### Identification of PT subtypes in the kidney

**Fig. 2:**
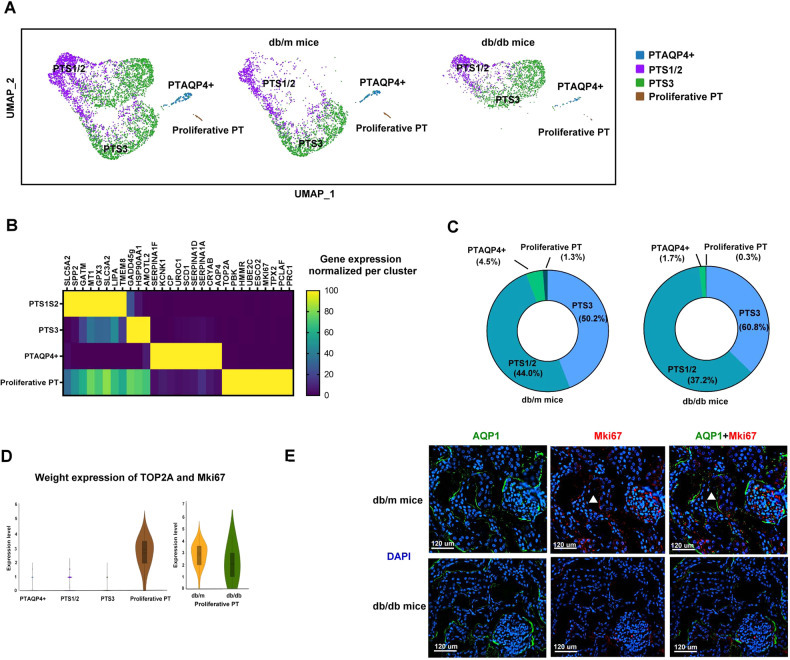
Proliferative PT modulated renovation function in the kidney. **A** Cell clusters of the PT of db/m mice and db/db mice. UMAP representation of the PT of db/m mice and db/db mice. **B** The specific markers of cell cluster of PT subtypes. **C** The percentage of each PT subtypes. **D** Weighted analysis of the expression of proliferative markers (TOP2A, MKi67) in four PT subtypes. **E** The kidneys of db/m mice and db/db mice were collected and staining by CODEX multiplexed tissue staining. PT (AQP1, green), proliferative PT (MKi67, red), DAPI (blue). Scale bars, 120 μm. ▲ (proliferative PT).

Four distinct clusters of PT with specific markers were identified after analysis using re-clustering (Fig. 2A). They were defined as PTS1/2, PTS3, PT containing AQP4 expression (PT^AQP4+^), and proliferative PT. Among the four PT subtypes, the up-expression of differentially expressed genes (DEGs) for PTS1/2 (SLC5A2, SPP2, GATM, MT1, GPX3, SLC3A2, LIPA, TMEM8), PTS3 (GADD45G, HSP90AA1, AMOTL2), PT^AQP4+^ (SERPINA1F, KCNK1, CP, UROC1, SCD1, SERPINA1D, SERPINA1A, CRYAB, AQP4), and proliferative PT (TOP2A, PBK, HMMR, UBE2C, ESCO2, MKi67, TPX2, PCLAF, PRC1) were shown in the corresponding cell clusters (Fig. 2B). The percentages of proliferative PT and PT^AQP4+^ cells were decreased in the kidneys of db/db mice compared to those in db/m mice (Fig. 2C).

### Proliferative PT modulated renovation function in the mice kidneys

Next, we focused on proliferative PT due to decreased cell number in db/db mice. Proliferative PT expressed classical cell proliferative markers including TOP2A and MKi67 compared to other PT subtypes (Fig. 2D). Immunofluorescence (IF) staining also revealed a decrease in the cell number of proliferative PT (AQP1^+^MKi67^+^) in db/db mice compared to db/m mice (Fig. 2E). Bioinformatics analysis revealed that proliferative PT modulated renal necrosis/cell death, cell cycle and P53 signaling (Supplementary Fig. 2A, Supplementary Table 1). The DEGs of the proliferative PT of db/db mice were associated with cell proliferation, differentiation, survival and DNA repair processes compared to db/m mice (Supplementary Fig. 2B, Supplementary Table 2). These findings suggest that the repair function of PT in early DKD could be caused by a decrease in proliferative PT cells.

### PT^AQP4+^ modulated renin-angiotensin system (RAS) in the mice kidneys

**Fig. 3:**
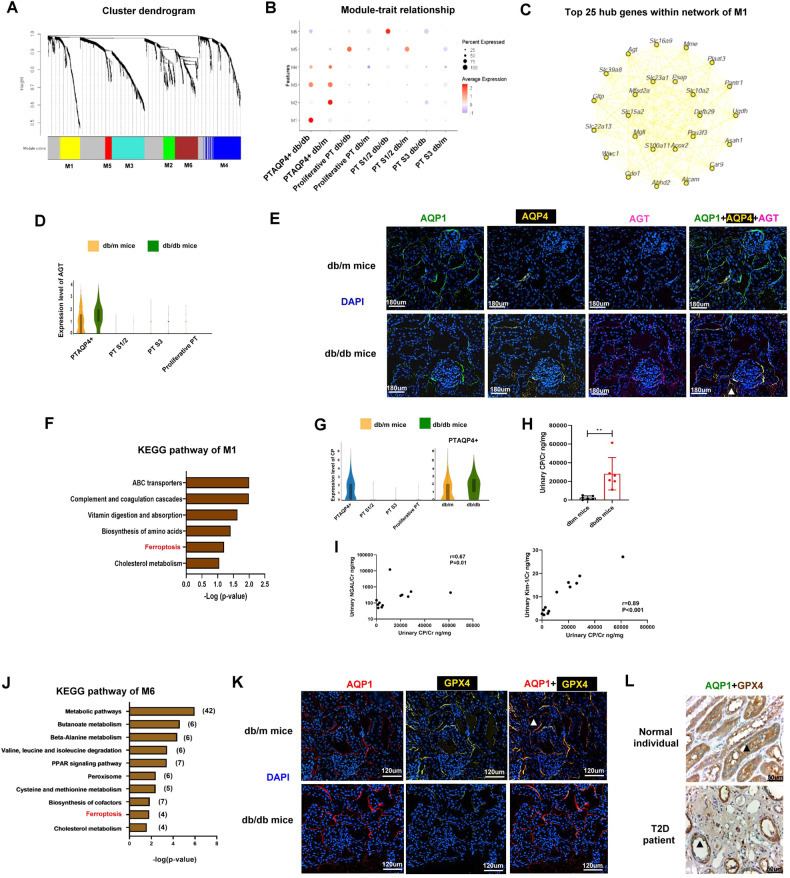
PT^AQP4+^ modulated RAS and ferroptosis play a pathophysiologic role in PT microenvironment of DKD. **A** WGCNA analysis of transcriptome of PT subtypes of mice. Each leaf (vertical line) in the dendrogram corresponds to a gene. **B** Module-trait relationship based on WGCNA analysis of PT subtypes of mice. **C** The network of module 1 of the WGCNA analysis. **D** Violin plot of AGT in PT subtypes of db/m and db/db mice. **E** The kidneys of db/m mice and db/db mice were collected and staining by CODEX Multiplexed Tissue Staining. PT (AQP1, green), PT^AQP4+^ (AQP4, yellow), AGT (pink), DAPI (blue). Scale bars, 180 μm. ▲ (triple stain of AQP1, AQP4 and AGT). **F** KEGG pathway analysis of module 1 of the WGCNA analysis. **G** Violin plot of CP in PT subtypes of db/m and db/db mice. **H** Urinary CP/Cr levels in db/m (*n* = 6) and db/db mice (*n* = 6). **I** The relationship between urinary CP/Cr and albuminuria, NGAL/Cr and Kim-1/Cr in mice. The level of CP, NGAL and Kim-1 in the urine measured using ELISA. Levels of urinary albumin and Cr assessed using the immunoturbidimetric assay and the enzymatic method respectively. **J** KEGG pathway analysis of module 6 of the WGCNA analysis. **K** The kidneys of db/m mice and db/db mice were collected and staining by CODEX multiplexed tissue staining. PT (AQP1, red), GPX4 (yellow), and DAPI (blue). Scale bars, 120 μm. **L** The kidneys of a normal individual and a type 2 diabetes (T2D) patient were collected and staining by Immunohistochemistry (IHC) stain. PT (AQP1, green) and GPX4 (brown). Scale bars, 50 μm. ▲ (double stain of GPX4 and AQP1). The bar graph represents the mean ± S.E.M. *p < 0.05, **p < 0.01, ***p < 0.001 by Student’s *t* test, and *p*-value of correlation was analyzed by Spearman analysis.

Another major change in PT in early DKD was a decrease in the number of PT^AQP4+^. To assess the key molecules regulated by various types of PT in DKD, we performed weighted gene co-expression network analysis (WGCNA) and the results showed that module 1 (M1) was positively correlated with PT^AQP4+^ in db/db mice, whereas module 6 (M6) was positively associated with PTS1/2 in db/db mice (Fig. 3A, B). The top five pathways of tox list for M1 included LXR/RXR and FXR/RXR signaling pathway based one Ingenuity Pathway Analysis (IPA) (Supplementary Fig. 3A). Among the top 25 hub genes of M1 within the interaction network according to WGCNA (Fig. 3C), angiotensinogen (AGT), as the key molecule in RAS, was associated with LXR/RXR and FXR/RXR signaling, and AGT was only expressed in PT^AQP4+^ but not in the other subtypes of PT. The expression of AGT was higher in db/db mice than db/m mice (Fig. 3D, Supplementary Table 3). Co-Detection by indEXing (CODEX) staining revealed a higher expression of AGT in PT of db/db compared to those of db/m mice (Fig. 3E). Their results suggested that PT^AQP4+^ may regulate RAS.

### Ferroptosis played a pathophysiologic role in PT microenvironment in DKD

Kyoto Encyclopedia of Genes and Genomes (KEGG) analysis of M1 revealed that the hub genes regulated by PT^AQP4+^ in db/db mice were associated with ferroptosis (Fig. 3F, Supplementary Table 4). Among the hub genes related to ferroptosis, ceruloplasmin (CP) was uniquely expressed in PT^AQP4+^ of db/db mice, but not in other types of PT (Fig. 3G). Furthermore, the level of urinary CP was higher in db/db mice than db/m mice (Fig. 3H), and it was positively correlated with PT injury markers kidney injury molecule-1 (KIM-1)/Cr and neutrophil gelatinase-associated lipocalin (NGAL)/Cr (Fig. 3I).

Ferroptosis was also found in the KEGG pathway of M6, indicating that the hub genes regulated by PTS1/2 in db/db mice were associated with ferroptosis (Fig. 3J, Supplementary Table 5). The tox list indicated that M6 was associated with NF-E2-related factor 2 (NRF2)-mediated oxidative stress response, which plays an important role in ferroptosis (Supplementary Fig. 3B, Supplementary Table 6). IF staining revealed lower levels of glutathione peroxidase 4 (GPX4), a marker of ferroptosis, in PT (AQP1) of db/db mice than those in db/m mice, and immunohistochemistry (IHC) stain also found decreased GPX4 level in the PT (AQP1) of the T2D patient compared to the normal individual (Fig. 3K, L). Taken together, these findings suggest that ferroptosis may play an important pathophysiologic role in PT injury, especially PT ^AQP4+^ and PTS1/2, with regard to the progression of DKD.

### Decreased TAL and dysregulated metabolism in collecting ducts (CDs) in early DKD

**Fig. 4:**
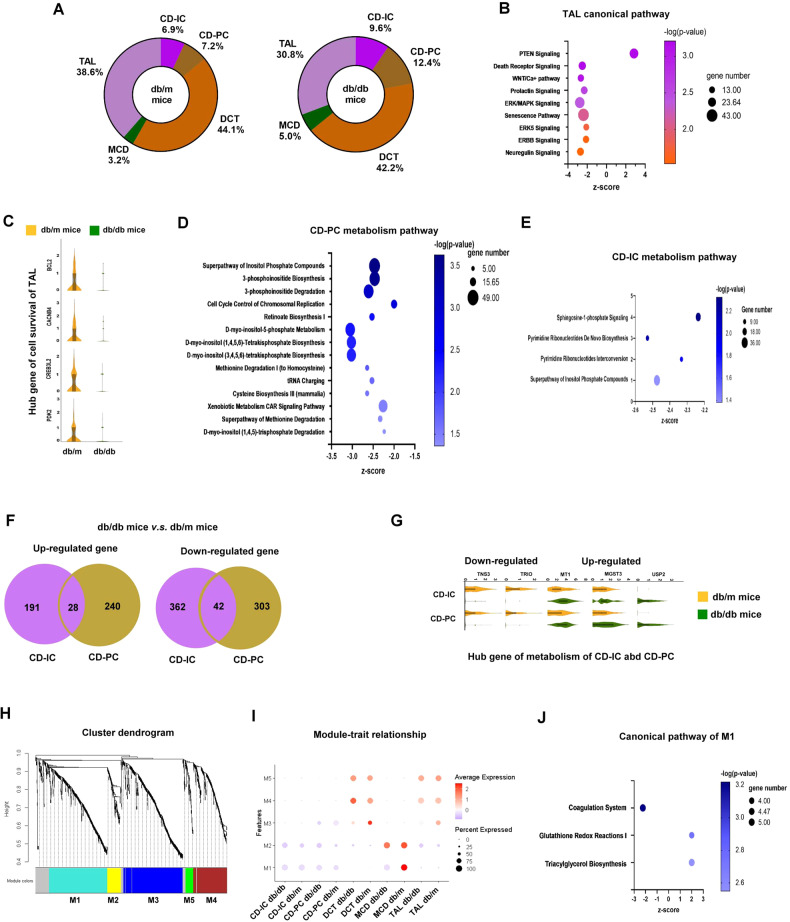
The decreased number of TAL and dysregulated metabolism in CDs in early DKD. **A** The percentage of the five tubular cells. **B** Canonical pathway of the gene of TAL of db/db mice compared to db/m mice. **C** Violin plot of the hub gene related to cell survival of TAL in db/m and db/db mice. **D**, **E** Canonical pathway related to metabolism regulation of CD-PC and CD-IC of db/db mice compared to db/m mice. **F** Venn diagram of the up-regulated and down-regulated hub genes between CD-IC and CD-PC of violin plot of db/db mice compared to db/m mice. **G** Up-regulated and down-regulated genes in CD-IC and CD-PC. **H** WGCNA analysis of five tubules. Each leaf (vertical line) in the dendrogram corresponds to a gene. **I** Module-trait relationship. **J** Canonical pathway of module 1 of the WGCNA analysis.

Fewer TAL and increased percentage of CD-IC and CD-PC were found in db/db mice compared to db/m mice (Fig. 4A). Functional pathway analysis of the DEG profile using IPA indicated that TAL in db/db mice was negatively correlated with cell survival-related signaling pathways (Fig. 4B, Supplementary Table 7). Several cell survival genes including BCL2, CACNB4, CREB3L2 and PDK2 were decreased in TAL of db/db mice, suggesting that cell death may contribute to the decrease in TAL in early DKD (Fig. 4C, Supplementary Table 7).

We found that the DEG profile of CD-PC in db/db mice was negatively correlated with several metabolism pathways, such as inositol phosphate, methionine, and cysteine biosynthesis and degradation (Fig. 4D, Supplementary Table 8). In addition, the hub genes of CD-IC in db/db mice were negatively associated with metabolism pathways, including sphingosine-1-phosphate and pyrimidine ribonucleotides biosynthesis (Fig. 4E, Supplementary Table 9). We used the Venn diagram to examine the potential pathophysiologic genes in CD-IC and CD-PC in DKD, which revealed 28 up-regulated genes and 42 down-regulated genes expressed in both CD-IC and CD-PC in db/db mice compared to db/m mice (Fig. 4F, Supplementary Table 10). Among these hub genes (Fig. 4G), USP2 and TNS3 were involved in inositol phosphate pathway in CD-IC and CD-PC. MGST3 participated in xenobiotic metabolism, and MT1 was associated with methionine degradation and cysteine biosynthesis in CD-PC of db/db mice (Supplementary Tables 8, 9).

We then performed WGCNA to explore the pathologic factors of TAL, DCT, and CD in DKD (Fig. 4H). The findings revealed that M1 was negatively correlated with MCD in the db/db mice (Fig. 4I). Both canonical pathway and KEGG analysis indicated that M1 was positively correlated with regulation of metabolism including glutathione redox reactions and triacylglycerol biosynthesis (Fig. 4J, Supplementary Fig. 4A, Supplementary Tables 11, 12). Among the top 25 hub genes of M1 within the interactional network (Supplementary Fig. 4B), MGST1 was involved in glutathione-related metabolism, and its expression was higher in MCD of db/m mice than db/db mice (Supplementary Fig. 4C). These findings suggest that the decrease in TAL and impaired metabolism functions in CDs (CD-IC, CD-PC and MCD) may contribute to tubular injury in early DKD.

### SPP1 modulated communication within PT subtypes and distal tubules in early DKD

**Fig. 5:**
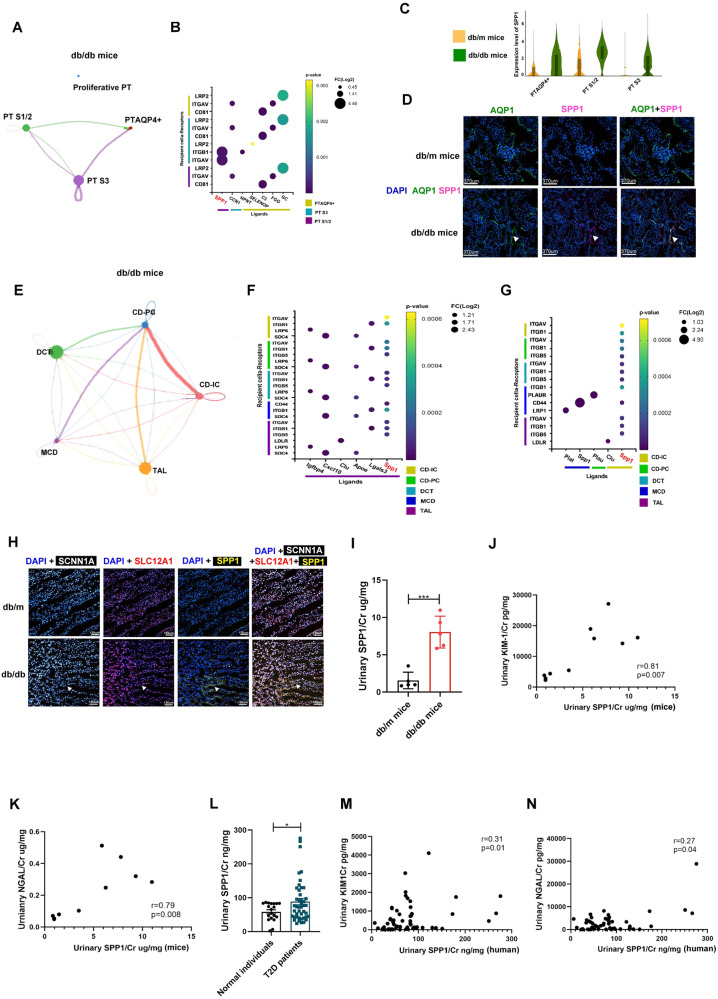
SPP1 modulates the communication within PT subtypes and distal tubules in early DKD. **A, B** The interaction among four PT subtypes in db/db mice. **C** Violin plot of SPP1 among PT subtype between db/m and db/db mice. **D** The kidneys of db/m mice and db/db mice were collected and staining by CODEX Multiplexed Tissue Staining. PT (AQP1, green), SPP1 (pink), and DAPI (blue). Scale bars, 370 μm. ▲ (double stain of SPP1 and AQP1). **E** The interaction among TAL, DCT, CD-IC, CD-PC and MCD in db/db mice. **F** The communication between TAL and other five types of kidney tubules in db/db mice. **G** The communication of MCD, CD-IC and CD-PC with other five types of kidney tubules in db/db mice. **H** The kidneys of db/m mice and db/db mice were collected and staining by CODEX multiplexed tissue staining. TAL (SLC12A1, red), CD-IC (SCNN1A, white), SPP1 yellow), and DAPI (blue) were indicated by the white arrow. Scale bars, 120 μm. ▲ (Triple stain of SPP1, SCNN1A, and SLC12A1). **I** Urinary SPP1/Cr levels in db/m (*n* = 6) and db/db mice (*n* = 6). **J, K** The relationship between urinary SPP1 and Kim-1/Cr and NGAL/Cr in mice. **L** Urinary SPP1/Cr levels in 24 normal individuals and 48 T2D patients. **M**, **N** The relationship between urinary SPP1 and Kim-1/Cr and NGAL/Cr in human. The level of SPP1, NGAL and Kim-1 in the urine measured using ELISA. Levels Cr assessed using the enzymatic method. **J** The level of SPP1 in the urine measured using ELISA. The bar graph represents the mean ± S.E.M. *p < 0.05, **p < 0.01, ***p < 0.001 by Student’s *t* test, and *p*-value of correlation was analyzed by Spearman analysis.

Potential interactions among the four PT subtypes were examined to clearly understand differences in PT microenvironment in DKD. We assessed the up-regulated ligand-receptor pairs with fold change (FC) > 2 in the PT subtypes. Compared to db/m mice, potential interactions between PT^AQP4+^ and the other PT subtypes (PTS1/2 and PTS3) including C3-CD81, FGG-ITGAV, and GC-LRP2 axis were only found in db/db mice (Fig. 5A, B; Supplementary Tables 13, 14). Among the hub genes involved in cross-talk among the PT subtypes in db/db mice, SPP1 had the highest expression and participated in communication between PTS1/2 and PTS3. Tox list analysis of the DEGs of PTS1/2 and S3 between db/db mice and db/m mice also revealed that SPP1 was involved in VDR/RXR activation and renal necrosis/cell death (Supplementary Tables 15, 16). SPP1 expression was elevated in PTS1/2, PTS3, and PT^AQP4+^ in db/db mice compared to db/m mice (Fig. 5C). IF staining revealed a higher expression level of SPP1 at the protein level in PT of db/db mice than db/m mice, meaning that SPP1 may regulate the pathophysiologic cross-talk among PT subtypes in early DKD (Fig. 5D).

We also examined communication among TAL, DCT, and CDs in DKD. In db/db mice (Fig. 5E), TAL uniquely communicated with other tubules through SPP1 signaling pathway (Fig. 5F, Supplementary Table 17). CD-IC also interacted with the other four tubular types through SPP1 signaling pathway. Interactions were potentially through SPP1-CD44 within MCD (Fig. 5G). Multiple IF was further performed, which revealed higher expression of SPP1 in TAL (SLC12A1^+^) and CD-IC (SCNN1A^+^) in db/db mice compared to db/m mice (Fig. 5H). db/db mice had a higher expression level of urinary SPP1/Cr than db/m mice (Fig. 5I), and urinary SPP1/Cr level was positively correlated with PT injury markers including NGAL/Cr and KIM-1/Cr (Fig. 5J, K). We further enrolled normal individuals and T2D to validate SPP1 as the biomarker of DKD (Supplementary Table 18). T2D patients had higher urinary SPP1/Cr level than normal individuals (Fig. 5L) and the positive relationship between urinary SPP1/Cr level and PT injury markers was also shown (Fig. 5M, N). These findings indicated that SPP1 not only plays an important role in communication within both the proximal and distal tubular microenvironment of DKD but also is a potential biomarker of tubular injury in early DKD.

### MC-secreted semaphorin 3C (SEMA3C) regulated cross-talk in glomerular microenvironment in early DKD through Neuropilin 1 (NRP1) and Neuropilin 2 (NRP2) pathway

**Fig. 6:**
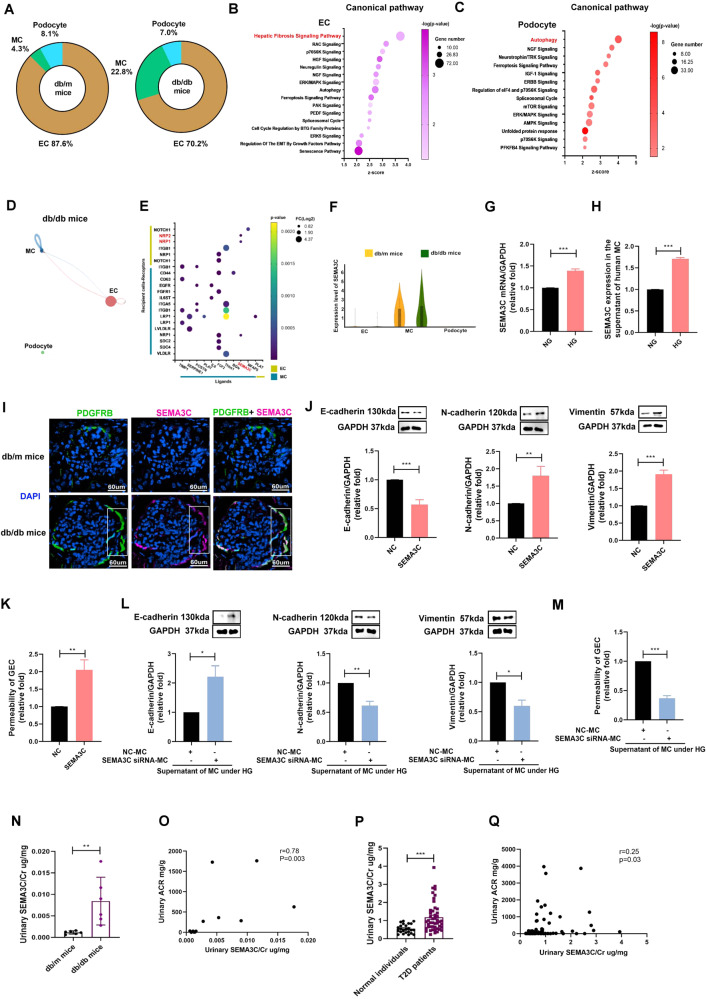
MC-secreted SEMA3C regulated the cross-talk of glomerulus microenvironment of early DKD. **A** The percentage of glomerular cells. **B**, **C** Canonical pathway of the gene of EC and podocyte of db/db mice compared to db/m mice. **D**, **E** The interaction within glomerulus in db/db mice. **F** Violin plot of SEMA3C in MC, EC and podocyte of db/m and db/db mice. **G** mRNA expression of SEMA3C in MC treated with NG and HG for 48 h measured by qRT-PCR (*n* = 3). **H** SEMA3C protein expression in the supernatant of MC treated with NG (5.5 mM) and HG (25 mM) for 48 h measured by ELISA. **I** The kidneys of db/m mice and db/db mice were collected and staining by CODEX Multiplexed Tissue Staining. MC (PDGFRB, green), SEMA3C (pink), and DAPI (blue). Scale bars, 60 μm. **J** E-cadherin, N-cadherin and vimentin expression in GECs treated with SEMA3C (10 ng/ml) for 48 h using western blotting (*n* = 3). **K** Permeability of GECs treated with SEMC3C (10 ng/ml) for 48 h using transendothelial permeability assay (*n* = 3). **L** MC was transfected with SEMA3C siRNA (20 nM) or NC (20 nM) for 24 h, and then treated under HG condition for 48 h. GEC was cultured with the supernatant of HG-treated MC for 48 h. E-cadherin, N-cadherin and vimentin expression in cultured GECs using western blotting (*n* = 3). **M** Permeability of cultured GECs using transendothelial permeability assay (*n* = 3). **N** Urinary SEMA3C/Cr levels in db/m (n = 6) and db/db mice (*n* = 6). **O** The relationship between urinary SEMA3C/Cr levels and urinary ACR in mice. **P** Urinary SEMA3C/Cr levels in 24 normal individuals and 48 T2D patients. **Q** The relationship between urinary SEMA3C/Cr levels and urinary ACR in human. The level of SEMA3C in the urine measured using ELISA. Urine albumin was measured using the immunoturbidimetric assay. Levels Cr assessed using the enzymatic method. The bar graph represents the mean ± S.E.M. *p < 0.05, **p < 0.01, ***p < 0.001 by Student’s *t* test or ANOVA followed by the post hoc test with Tukey’s correction, and *p*-value of correlation was analyzed by Spearman analysis.

Compared to db/m mice, lower percentages of podocyte and EC and a higher percentage of MC were found in db/db mice (Fig. 6A). Functional pathway analysis with IPA revealed that the DEGs of EC were positively associated with fibrosis signaling while the DEG profile of podocyte was correlated with autophagy and cell survival and death (Fig. 6B, C; Supplementary Tables 19, 20).

With regards to the unique interactions in the glomerular microenvironment of db/db, mice (Fig. 6D), MC potentially communicated with EC though the SEMA3C- NRP1/NRP2 axis (Fig. 6E, Supplementary Table 21). In addition, the SEMA3C-NRP1 axis was involved in the interaction within MCs. The expression of SEMA3C was higher in MC of db/db mice than db/m mice (Fig. 6F). An in vitro study validated that high glucose (HG) increased SEMA3C mRNA expression in MC (Fig. 6G). In addition, elevated SEMA3C protein secretion was found in the supernatant of MC cultured under HG (Fig. 6H). IF staining confirmed higher levels of SEMA3C in MC (PDGFRB^+^) of db/db mice than db/m mice (Fig. 6I). SEMA3C promoted endothelial–mesenchymal transition (EndoMT) and increased permeability in glomerular endothelial cell (GEC, Fig. 6J, K), but did not change cell viability (Supplementary Fig. 5A). After silencing SEMA3C by siRNA transfection in MC under HG (Supplementary Fig. 5B), EndoMT and increased permeability in GEC induced by the supernatant of HG-stimulated MC were prevented (Fig. 6L, M). We further identified whether MC-secreted SEMA3C contributed to GEC injury, and the results found that blocking NRP1 or NRP2 by neutralizing antibody (nAb) ameliorated EndoMT and the increase in permeability in GEC induced by the supernatant from HG-stimulated MC (Supplementary Fig. 5C–F). Similarly, suppression of NRP1 or NRP2 alleviated SEMA3C-induced GEC injury including EndoMT and increased permeability (Supplementary Fig. 5G–J). Furthermore, urinary SEMA3C/Cr level were higher in db/db mice than db/m mice, and urinary SEMA3C/Cr level was positively associated with urinary ACR in mice (Fig. 6N, O). These consistent results were also found in human (Fig. P, Q). Our findings demonstrated that SEMA3C participates in cross-talk between MC and GEC, and MC secretes SEMA3C to induce GEC injury through NRP1/NRP2 in DKD. SEMA3C has the potential to be a therapeutic target or biomarker of early DKD.

## Discussion

**Fig. 7:**
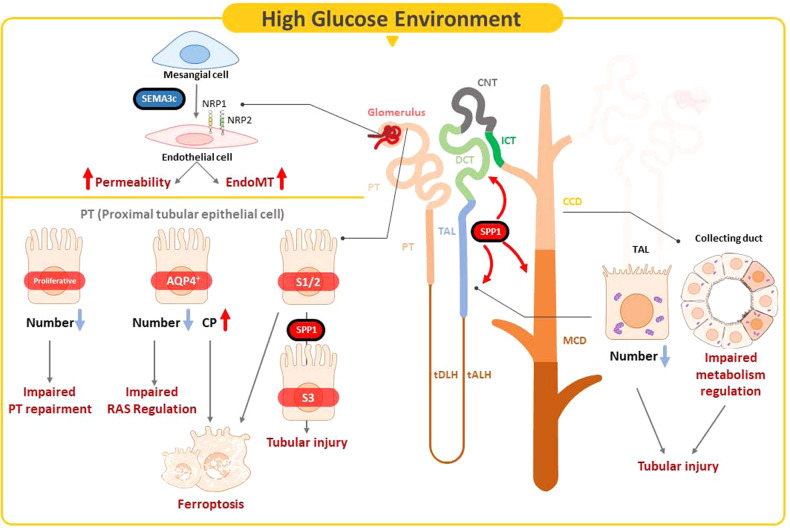
Illustration of the mechanism of the hub gene contributing to the pathogenesis of early DKD. In early DKD, lower percentages of proliferative PT and PT^AQP4+^ induced impaired cell repair activity and dysfunction of RAS modulation. CP acted as a central regulator of the induction of ferroptosis in PT^AQP4+^. Lower percentages of TAL and CD with impaired metabolism function were pathogenic features. SPP1 mediated pathogenic cross-talk in the tubular microenvironment. MC-derived SEMA3C further promoted endothelium-mesenchymal transition in GEC through NRP1 and NRP2 pathway.

DKD is a major cause of ESRD worldwide; however, the complex pathophysiologic mechanisms of DKD, especially at the early stage, are not well-understood at the single-cell transcriptional level. The gap in knowledge hinders the development of precise diagnostic biomarkers or treatment strategies. In this study, we generated the single-cell transcriptome atlas for early stage of DKD, and further used in vitro, in vivo and human setting to prove the pathophysiologic role of hub genes. We demonstrated two novel subtypes of PT, namely proliferative PT and PT^AQP4+^. We also found that the pathophysiologic role of proliferative PT was different compared to the other three PT subtypes. PT^AQP4+^ may be the major subtype of PT to modulate RAS, and ferroptosis participated in PT^AQP4+^ and PTS1/2 injury in early DKD. Urinary levels of CP, which is involved in ferroptosis, were positively associated with PT injury. SPP1 mediated the pathophysiologic cross-talk in the tubular microenvironment in early DKD. In addition, MC communicated with EC through SEMA3C, further promoting EC injury through NRP1 and NRP2 pathway. Urinary SEMA3C level was also the potential biomarker of glomerular injury in early DKD. Our findings identified the hub genes involved in pathophysiologic changes within the glomerular and kidney tubular microenvironment in early DKD (Fig. 7).

PT cells are numerically the predominant constituent of the kidney, and play an important role in the progression of DKD [14]. We identified four subtypes of PT, of which proliferative PT and PT^AQP4+^ were novel in db/m and db/db mice. The pathophysiologic role of proliferative PT was different from the other three PT subtypes. Proliferative PT expressed significant proliferative gene signatures including PROM1, TOP2A and MKi67, which have stem/progenitor properties and were can differentiate into multiple lineages, indicating its role in cell proliferation [15]. PROM1 has been demonstrated to be able to repair renal tubular injury through its regenerative potential [15, 16]. MKi67 and TOP2A have been shown to modulate DNA replication of cell cycle, and participate in renal repair after acute kidney injury [17]. We found the lower cell numbers and expression of the genes associated with progenitor properties of proliferative PT in db/db mice compared to db/m mice, meaning that DKD may result in the loss of the ability to repair damage in PT. In addition, proliferative PT may transmit signals of cell proliferation or anti-apoptosis to preserve kidney structure or function. However, proliferative PT did not interact with other PT populations in our DKD model, probably due to the limited numbers of proliferative PT in db/db mice.

Another unique finding of this study is the identification of demonstrate the new PT population, PT^AQP4+^ and its pathophysiologic role in DKD. AQP4 usually presents in basolateral plasma membrane of principal cells of the CD. Previous studies have reported the presence of AQP4 in PTS3 [18, 19], but without clarifying its physiologic role in kidney. We found a significant difference between PT^AQP4+^ and PTS3 owing to the unique genes expressed in PT^AQP4+^. Distinct from the other PT subtypes, the genes of PT^AQP4+^ were closely correlated with RAS. Since the role of RAS in kidney dysfunction in early 1990s, inhibitors of angiotensin-converting enzyme and blockers of angiotensin II type 1 receptors have been the standard therapy for DKD [20]. Over-activation of RAS induces intracapillary hypertension and hyper-ultrafiltration in kidney, leading to kidney injury. AGT is the key molecule in RAS activation and produced in the PT cells [21]. Our study shows that AGT participated in RAS pathway dominantly in PT^AQP4+^, and a higher expression of AGT was found in PT^AQP4+^ in db/db mice. While scRNA-seq technology provides a new level of data resolution, it also contributes to a larger number of genes reporting the expression level to be zero or practically zero, as compared to using bulk RNA-seq. It is probably related to either biologically driven, genes not expressing RNA at the time of measurement, or technically driven, genes expressing RNA without a sufficient level to be detected by sequencing technology [22]. Thus, the current results may indicate that PT^AQP4+^ is the major target of PT subtypes in RAS activation in DKD.

We also performed WGCNA of PT subtypes, and the results revealed that ferroptosis was an important pathophysiologic mechanism in early DKD, especially in PTS1/2 and PT^AQP4+^. Ferroptosis is characterized by iron-catalyzed intracellular accumulation of lipid hydroperoxides, and it is a distinct non-apoptotic form of regulated cell death [23]. Wang et al. have found that tubulouinterstitial GPX4 expression level, a marker of ferroptosis, was correlated with human DKD progression using IHC stain [24]. Liu et al. used the Affymetrix® microarray platform to identify ferroptosis-related genes and pathways in DKD [25]. However, the pathophysiologic role of ferroptosis on different types of PT is not defined. Owing to the distinct impact of PT subtypes on pathophysiology of DKD, an exactly exploration of ferroptosis on PT subtypes is needed. Our study examined the features of ferroptosis within different PT subtypes, and found a lower expression of GPX4 in PTS1/2 and PT^AQP4+^ in db/db mice compared to db/m mice. Ferroptosis was especially dominant in PTS1/2 and PT^AQP4+^, and less expressed in PTS3 or proliferative PT in early DKD. Furthermore, among the hub genes of PTS1/2 and PT^AQP4+^ involved in ferroptosis, the level of CP in urine could be a biomarker of kidney injury in DKD. These findings provide the predictive value of urinary CP level from single-cell transcriptomes to in vivo, suggesting a potential pathophysiologic role of CP in PT injury through ferroptosis in early DKD.

TAL, DCT and CD play a principle role in the regulation of electrolyte, solute, and water reabsorption and excretion. Compared to glomerulus or PT, less research has investigated the role of these kidney tubules in the pathogenesis of DKD. Only a few reports have indicated that DKD may be associated with increased activity and expression of epithelial Na+ channels and sodium-hydrogen exchangers, which enhance sodium and volume reabsorption and exacerbate renal blood pressure [26]. The balance of potassium, calcium, and magnesium has also been correlated with the progression of diabetic complications [26]. A novel finding of our study is that the decreased number of TAL and impaired metabolic regulation functions in CDs in early DKD. Further investigations are necessary to identify the pathophysiologic role of TAL and CDs in early DKD.

Whether cross-talk among kidney tubules affects early DKD has yet to be clarified. Our findings revealed that SPP1 may participate in a complex mechanistic network related to the onset or progression of DKD, and that it could be a biomarker of tubular injury in early DKD in both in vivo and human models. SPP1 has been shown to modulate biological functions such as inflammation, immunologic response, and cell adhesion and migration [27]. Nicholas et al. demonstrated that SPP1 participated in the pathogenesis of mesangial expansion and albuminuria in DKD [28]. Further, reported that SPP1 could modify podocyte biology and promote TGF-β and MCP-1 production to increased mesangial matrix accumulation through binding to integrin αvβ3 [28]. Even though SPP1 has been demonstrated to be a predictive biomarker of DKD in a clinical setting [29], the pathophysiologic impact of SPP1 from PT to CD on the onset or progression of DKD remains unknown. An important and novel finding of the present study is that SPP1 may coordinate the interconnection among kidney tubules from proximal to distal nephrons.

Glomerular injury is a major characteristics of the pathogenesis of DKD [30]. Nevertheless, intraglomerular communication is still not well-established in DKD. In this study, we found that db/db mice lost interaction related to kidney development, and rather MC had unique interactions with GEC through SEMA3C-NRP1/NRP2 pathway. SEMA3C is a secreted glycoprotein, which may promote microvascular permeability in acute kidney injury [31]. Our results demonstrated that HG promoted SEMA3C secretion from MC, and MC-secreted SEMA3C induced Endo-MT and increased permeability of GEC through NRP1 or NRP2, meaning that communication between MC and EC may lead to EC damage in early DKD. We further demonstrated that suppressing SEMA3C could ameliorate GEC injury resulted from HG-stimulated MC, and urinary SEMA3C level was a biomarker of glomerular injury in early DKD. Therefore, SEMA3C has the potential for diagnosis and therapeutic target of early DKD.

Despite the tremendous development of scRNAseq technology, cell isolation and individualized RNA capture remain challenging. Even though enzymatic dissociation protocols usually compromise cell viability, the possibility of selective cell loss, especially glomerular cells, during tissue dissociation and the transcriptional stress response induced by the proteolytic process as well as RNA degradation contribute to bias [6, 32, 33]. In this study, the numbers of glomerular cells including podocyte, MC and EC were relatively low compared to kidney tubules in both db/m and db/db mice. Enzymatic digestion may damage podocyte whereas MC are less effectively isolated and captured due to the increased matrix based on DKD progression. Some researchers have recommended that cold dissociation with digestion on ice can avoid stress and achieve more abundant cell types than warm dissociation at 37^◦^C has been recommended by some researchers [34, 35]. In addition, we did not analyze the expression and mechanistic link of immunologic cells in DKD in this study because of limited inflammatory response in early DKD.

In conclusion, we identified the hub genes participating in the pathophysiology of early DKD and cross-talk of signaling transmission in early DKD. Our results provide not only novel insights into the mechanisms of DKD, but also potential biomarkers which could be used clinically to detect or trace DKD.

## Materials and Methods

### Experimental animals of early DKD

Five-week-old, pathogen-free male db/m and db/db mice were obtained from the National Laboratory Animal Center in Taiwan. All samples including blood, urine and kidneys were collected on the 14th, 22th and 33th week. The kidneys were fixed in 4% paraformaldehyde for immunochemistry staining. All animal experiments in this study were approved by Kaohsiung Medical University Animal Care and Use Committee.

### scRNA profiling, identification, and annotation of cell clusters

The scRNA sequencing data of three db/m mice and three db/db mice in the 14th week contained 6775 cells (3605 cells from db/m mice and 3170 cells from db/db mice). All analyses of the scRNA profiles were conducted following the standard workflow provided by the Seurat package (version 4.1.0, https://satijalab.org/seurat/) in R (version 4.0.4). The data were filtered according to the following thresholds: <200 or >2,500 as unique expressed genes (nFeature_RNA) and >30% as the percentage of mitochondrial genome content. The data were then normalized by conversion with a scale factor (default value of 10,000) and log-transformed using the Seurat embedded function. A correlation analysis and principal component analysis were performed and uniform manifold approximation and projection (UMAP) was used to classify cells into different cell clusters at a proper resolution.

Cell cluster annotation was conducted based on the known biomarkers of different cells in the kidney as published in previous studies (Supplementary Table 22) [8, 33]. DEGs among different clusters were identified to validate the reasonableness of cell cluster annotation.

### Weighted gene co-expression network analysis (WGCNA) of gene expression profile

We then investigated key molecules specifically expressed in kidney tubules in DKD by analyzing single-cell profiles. We identified potential hub genes involved in kidney tubules using WGCNA with the hdWGCNA R package (v0.1.1.9002) (https://github.com/smorabit/hdWGCNA). According to the instructions of this package, one-step network construction and module detection were used; the module eigengene expression, adjacency matrix heatmap and other related parameters/results were calculated and visualized.

### Identification of potential hub genes related to DKD using enrichment and DEG analyses

Enrichment analysis was performed to clarify the biological functions of hub genes in DKD. Gene ontology (GO) enrichment was conducted on genes using the updated DAVID Bioinformatics Resources (https://david.ncifcrf.gov/). In addition, biologic functions of the hub genes were also analyzed using Ingenuity Pathway Analysis (IPA) software (Ingenuity Systems, Redwood City, CA, USA), which provides core analysis and toxicity lists of DEGs to identify potential pathogenetic pathways.

### Cell-cell communication

A database of known cytokine/chemokine receptor and ligand pairs was constructed using combined information from CellTalkDB (http://tcm.zju.edu.cn/celltalkdb/), CellPhoneDB (https://www.cellphonedb.org/), CellChart (v1.1.1) (https://github.com/sqjin/CellChat), and resources from previously published studies [36, 37]. This database was then filtered for possible receptor-ligand interactions using the following criteria: 1) each receptor or ligand should be expressed in at least 50% of the cells of an individual cluster; 2) fold-change of each ligand ≥ 2 compared to db/m mice; 3) each pair should be expressed within the secretory or recipient cells.

### Cell lines and cell culture

Human kidney-2 (HK-2) cells (ATCC^*®*^CRL-2190) were cultured in keratinocyte serum-free medium (Catalog 17005-042) plus 2% fetal bovine serum (FBS). Human GEC (Catalog 4000, ScienCell, Carlsbad, CA, USA) were cultured in endothelial cell medium (Catalog 1001) plus 5% FBS. Human renal MCs (Sciencell, Catalog 4200) were cultured in MC medium (Catalog 4201) with normal glucose (NG, 5.5 mM) supplemented with 5% FBS. Cells were treated with NG, HG, SEMA3C (10 ng/ml), NRP1 nAb (5 μg/ml), or NRP2 nAb (20 μg/ml) for the indicated times.

### Human study participants

Forty-eight T2D patients and 24 healthy volunteers of similar age from communities were invited. Demographic and medical data were obtained from medical records and interviews with patients. Blood and urine samples were taken after a 12 h fast for biochemistry studies at enrollment, and stored in a −80 °C freezer. Kidney sections were obtained from one DKD patient scheduled for biopsies and one upper tract urothelial carcinoma (UTUC) patient receiving nephrectomy. This study was approved by the Institutional Review Board (IRB) of Kaohsiung Medical University Hospital (KMUHIRB-G(I)-20150044, KMUHIRB-G(I)20160036, and KMUHIRB-20130089). All participants provided written informed consent in accordance with the Declaration of Helsinki.

### Enzyme-linked immunosorbent assay (ELISA) and measurement of urinary biomarkers of kidney injury

Levels of KIM-1, NGAL, CP, and SPP1, and SEMA3C in the urine of mice and human and expression in the supernatant of MC treated with NG or HG were measured using ELISA kits (Supplementary Table 23). Levels of urinary albumin were assessed using the with Tina-quant Albumin Gen.2 immunoturbidimetric assay (ALBT2, Roche, USA). Concentrations of urine Cr were examined using the enzymatic method (Roche Diagnostics, Mannheim, Germany). Concentrations of urinary albumin, KIM-1, NGAL, CP, SPP1, and SEMA3C were corrected by urine Cr before statistical analysis.

### RNA isolation, reverse transcription, and quantitative real time PCR (qRT-PCR)

Total RNA from cells was isolated using TRIzol Reagent (Life Technologies). Relative mRNA expression level of SEMA3C in cells was normalized to an internal control GAPDH. Relative expressions were presented using the 2^-ΔΔCt^ method. The primers sequences for qRT-PCR were SEMA3C (Forward, 5′-TCTTA AAGGCGAGGCTGGTG-3′; Reverse, 5′-TACACACACACGGCTGATCC-3′) and GAPDH (Forward, 5′-GAGTCAACGGATTTGGTCGT-3′; Reverse, 5′-TTGATT TTGGAGGGATCTCG-3′).

### Transient transfection

SEMA3C siRNA (20 nM) and NC siRNA (20 nM) (Supplementary Table 23) were transfected into cells using Lipofectamine 3000 transfection reagent (ThermoFisher Scientific) following the manufacturer’s protocols.

### Western blot analysis

The total protein of cells was extracted using RIPA (radio-immunoprecipitation assay) lysis buffer (EMD Millipore, USA). The denature protein was separated by 9–11% SDS-PAGE electrophoresis, and then transferred onto a PVDF membrane followed by blocking and immunoblotting with specific primary and secondary antibodies. Antibodies against N-cadherin, vimentin, and E-cadherin from BD Biosciences (NJ, USA) and GAPDH antibody from Millipore (Burlington, USA) were obtained (Supplementary Table 23). The blot signals were captured using a Proteinsimple+Fluorchem Q system (Alpha Innotech, USA). Densitometry of the blots was calculated using Image J software (USA).

### Permeability analysis

Transendothelial permeability was assessed using an In Vitro Vascular Permeability Assay kit (EMD Millipore, MA, USA). HGECs grown to confluence on collagen-coated inserts were exposed to NG, HG, SEMA3C (10 ng/ml), NRP1 nAb (5 μg/ml), NRP2 nAb (20 μg/ml) or normal control (NC) for 48 h. After treatment, FITC-labeled dextran was added to the top of the cell monolayer for 20 min, and then FITC-dextran penetrated from the GEC monolayer to the bottom of the wells was measured by relative fluorescence excitation at 485 nm and emission at 530 nm using a fluorescence plate reader.

### Cell viability assay

The viability of cells was determined using a WST-1 Cell Proliferation Assay (ClontechTM Laboratories Inc., Mountain View, CA, USA). GECs (7 × 10^3^ cells/well) were seeded in 96-well plates and treated with different doses of SEMA3C (10 ng/ml) for 48 h. Cultured cells were incubated with WST-1 reagent at 37°C for 1 h, and absorbance at 450 nm was detected using an ELISA reader.

### Co-Detection by indEXing (CODEX) staining and imaging of the mice kidneys

Barcode-conjugated antibody staining of tissue sections mounted on polylysine-coated cover slips was performed using a commercially available CODEX Staining Kit with fresh frozen tissue using a Akoya CODEX instrument (Akoya Biosciences, Marlborough, MA, USA) with a Keyence BZ-X800 microscope plus Nikon PlanApo NA 0.75 objective (Nikon, Tokyo, Japan). Images were acquired at ×20 magnification and filter cubes for TRITC (550), CY5 (647), and CY7 (750) complementary fluorescent reporters and DAPI (4′,6-diamidino-2-phenylindole). Typical images were 3 × 3 mm including acquisition of five Z-stack images. The images were collected using CODEX Processor software (version 1.30.0.12). The antibodies used in CODEX staining are listed in Supplementary Table 23.

### Immunohistochemistry stain (IHC) stain

The features of kidney were detected using Periodic acid–Schiff (PAS) stain according to the manufacturer’s protocols (Sigma). Antibodies of GPX4 and AQP1 were used in IHC stain to detect ferroptosis in PT of kidney. Stained kidneys were observed using a Leica microscope (ICC50 HD, USA) and quantification was performed using the IHC Profiler Plugin of ImageJ Software (http://imagej.org/).

### Statistical analysis

Statistical analysis was performed using Prism version 9.2 (GraphPad Software). One-way analysis of variance was used to identify differences between two or more experimental groups, and Student’s unpaired *t* tests were used for comparisons between two treatment groups. All results are presented as mean ± standard error of the mean (SEM), and *P* values of <0.05 were considered statistically significant.

## Supplementary information

Supplementary Figures and Tables

Legends of supplementary Figures and Tables

Supplementary Table 1

Supplementary Table 2

Supplementary Table 3

Supplementary Table 4

Supplementary Table 5

Supplementary Table 6

Supplementary Table 7

Supplementary Table 8

Supplementary Table 9

Supplementary Table 10

Supplementary Table 11

Supplementary Table 12

Supplementary Table 13

Supplementary Table 14

Supplementary Table 15

Supplementary Table 16

Supplementary Table 17

Supplementary Table 18

Supplementary Table 19

Supplementary Table 20

Supplementary Table 21

Supplementary Table 22

Supplementary Table 23

Reproducibility checklist

## Data Availability

The data that support the findings of this study are available from the corresponding author upon reasonable request.
