# Supplementary figures and images for: Single-cell transcriptomic profiles in the pathophysiology within the microenvironment of early diabetic kidney disease
Source: Cell Death Dis. 2023 Jul 17;14(7):442. doi: 10.1038/s41419-023-05947-1 (PMC10352247; doi:10.1038/s41419-023-05947-1)

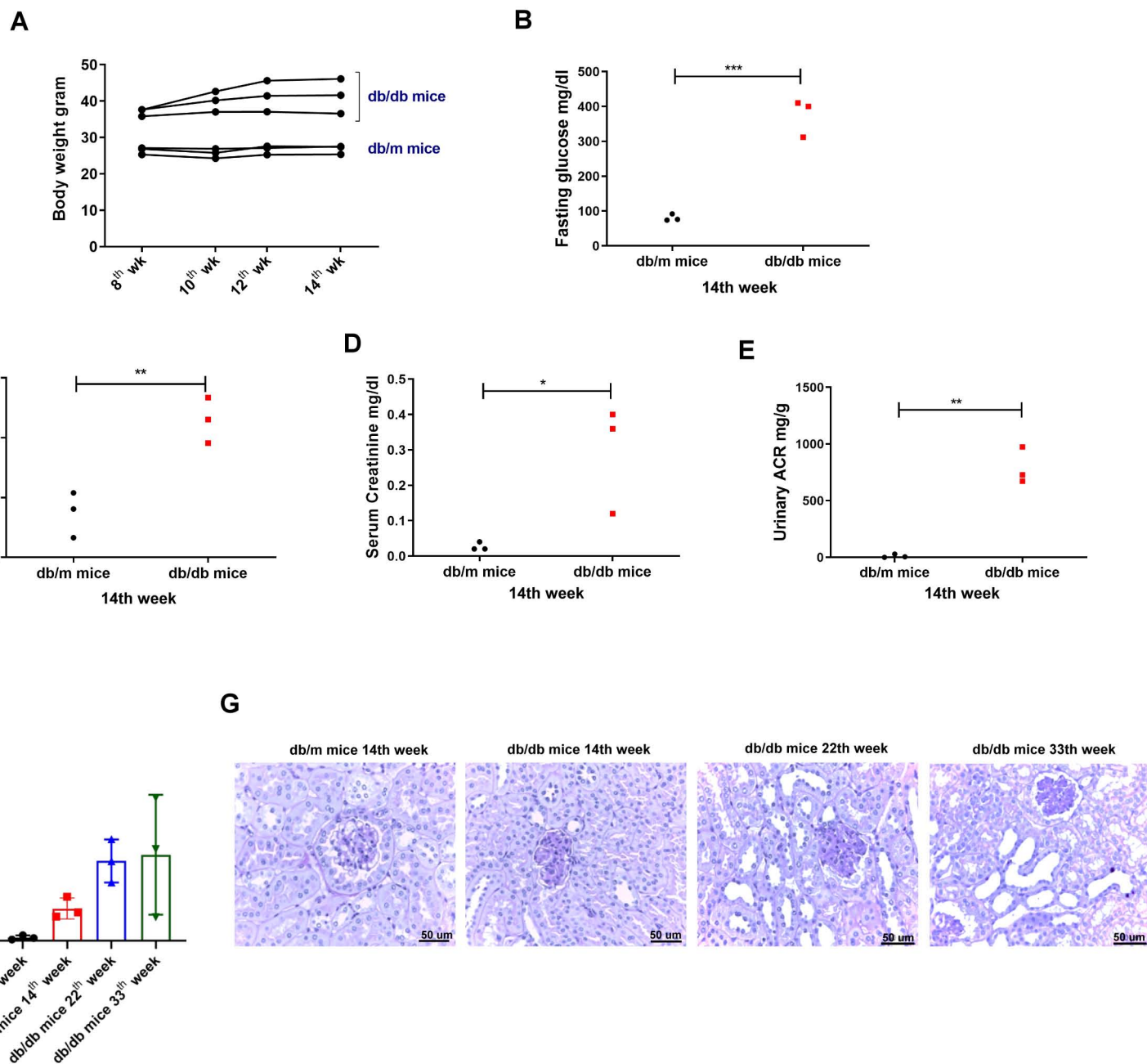

Fig S1

A

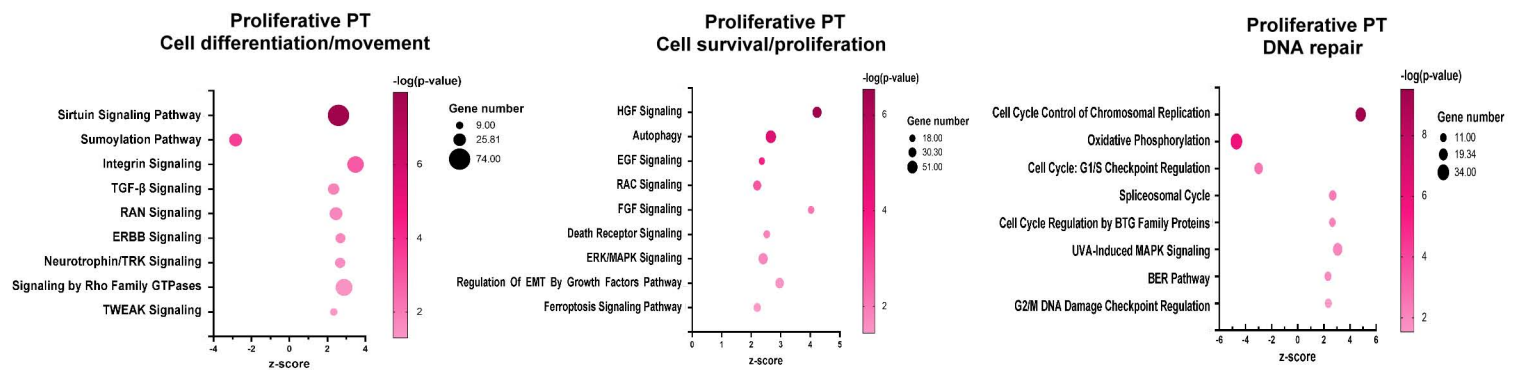

B

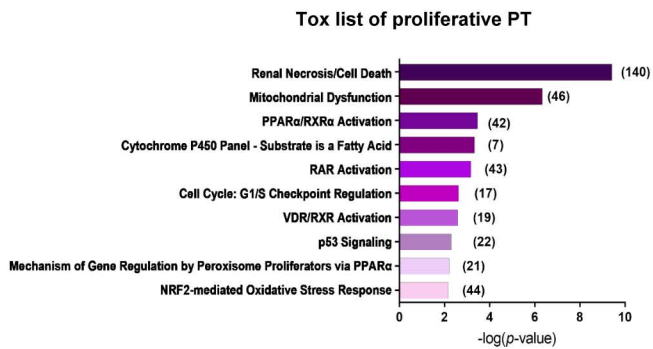

Fig S2

**A**

**Tox list of M1**

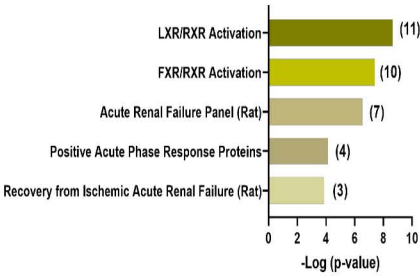

**B**

**Tox list of M6**

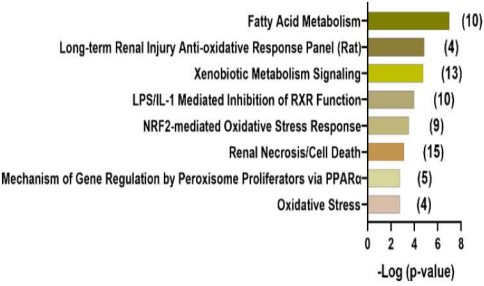

**Fig S3**

**A**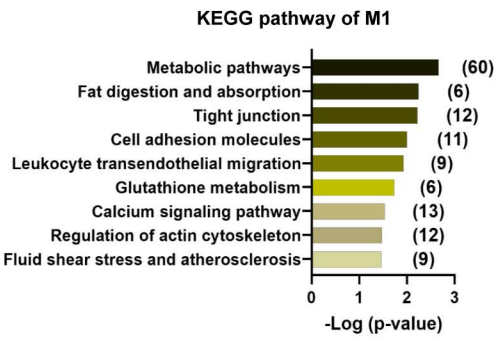**B**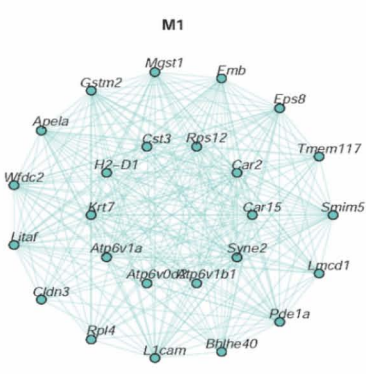**C**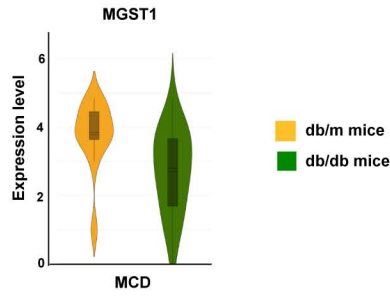

Fig S4

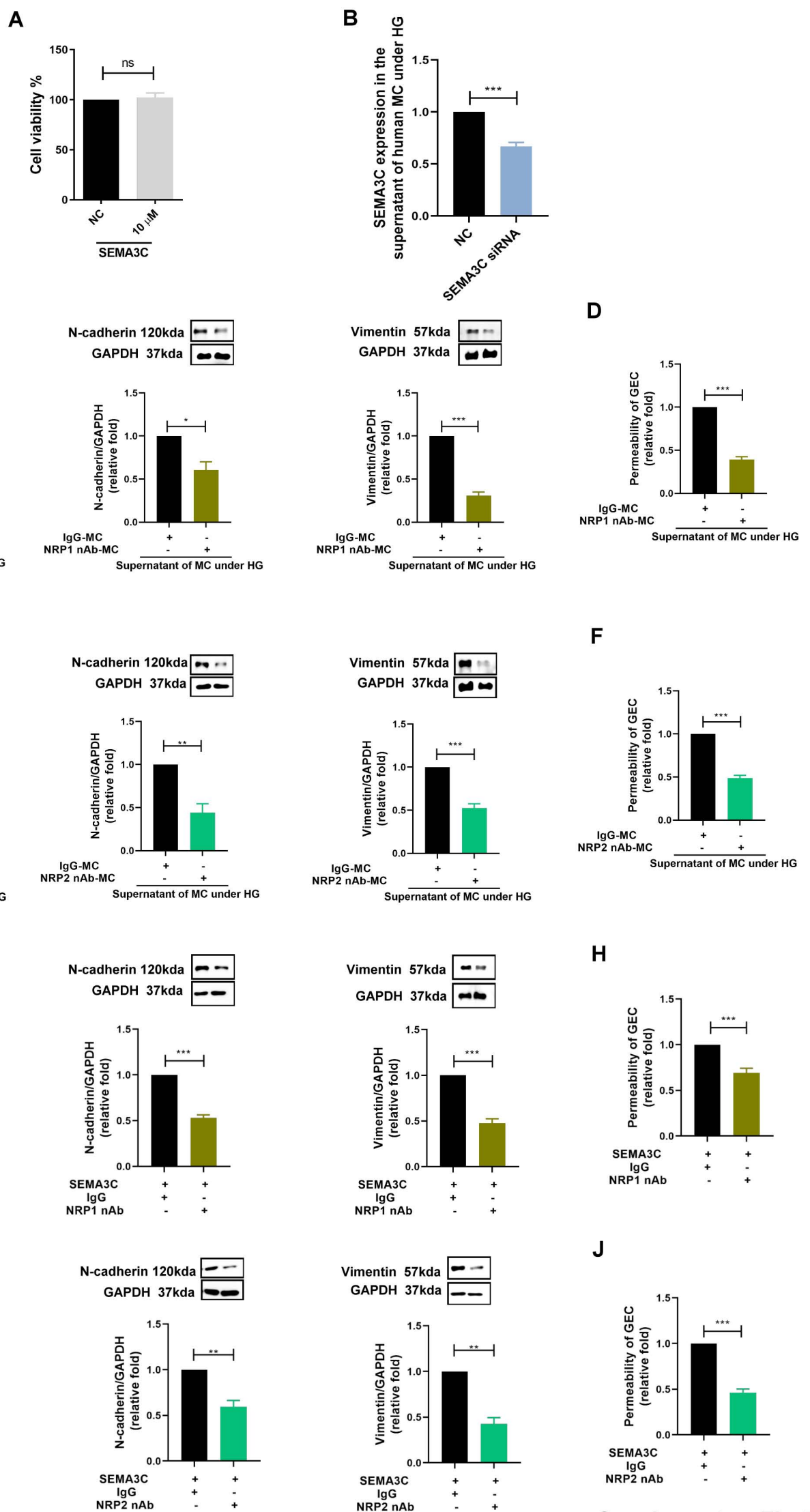

Supplementary Fig 5

Supplement: Supplementary file 1 — Supplementary Figures and Tables [file 41419_2023_5947_MOESM1_ESM.pdf]
