# Supplementary material for: Single-cell transcriptomic profiles in the pathophysiology within the microenvironment of early diabetic kidney disease
Source: Cell Death Dis. 2023 Jul 17;14(7):442. doi: 10.1038/s41419-023-05947-1 (PMC10352247; doi:10.1038/s41419-023-05947-1)
Supplement: Supplementary file 2 — Legends of supplementary Figures and Tables [file 41419_2023_5947_MOESM2_ESM.docx]

**Supplementary materials:**

**Supplementary Table 1** The tox list of the DEGs of proliferative PT in db/db mice compared to db/m mice

**Supplementary Table 2** The canonical pathway of the DEGs of proliferative PT in db/db mice compared to db/m mice

**Supplementary Table 3** The tox list of module 1 (PT^AQP4+^ in dbdb mice)

**Supplementary Table 4** The KEGG analysis of module 1 (PT^AQP4+^ in db/db mice)

**Supplementary Table 5** The KEGG pathways of module 6 (PTS1/2 in db/db mice)

**Supplementary Table 6** The tox list of module 6 (PTS1/2 in db/db mice)

**Supplementary Table 7** The canonical pathway of the DEGs of TAL in db/db mice compared to db/m mice

**Supplementary Table 8** The canonical pathway of the DEGs of CD-PC in db/db mice compared to db/m mice

**Supplementary Table 9** The canonical pathway of the DEGs of CD-IC in db/db mice compared to db/m mice

**Supplementary Table 10** Venn diagram of the hub genes of CD-IC and CD-PC in DKD

**Supplementary Table 11** The canonical pathway of module 1 (MCD in db/m mice)

**Supplementary Table 12** The KEGG pathway of module 1 (MCD in db/m mice)

**Supplementary Table 13** The interaction network of PT subtype in db/m mice

**Supplementary Table 14** The interaction network of PT subtype in db/db mice

**Supplementary Table 15** The tox list of the DEGs of PTS1/2 in db/db mice compared to db/m mice

**Supplementary Table 16** The tox list of the DEGs of PTS3 in db/db mice compared to db/m mice

**Supplementary Table 17** The interaction network of other tubules in db/db mice

**Supplementary Table 18** The characteristics of human participants in the study

**Supplementary Table 19** The canonical pathway of the DEGs of EC in db/db mice compared to db/m mice

**Supplementary Table 20** The canonical pathway of the DEGs of podocyte in db/db mice compared to db/m mice

**Supplementary Table 21** The interaction network of glomerulus in db/db mice

**Supplementary Table 22** Surface marker of kidney cells

**Supplementary Table 23** The antibodies used in study and sequence of SEMA3C siRNA

**Supplementary Fig 1.** Biochemistry data of db/m and db/db mice at the 14^th^ week and db/db mice at 22^th^ and 33^th^ week. **A-E** body weight, fasting glucose, blood urea nitrogen, serum creatinine, and urinary ACR. **F** Urinary albumin/creatinine ratio (ACR) at different ages of db/db mice. Urine albumin was measured using the immunoturbidimetric assay. Levels Cr assessed using the enzymatic method. **G** The features of the kidneys of different age of db/db mice assessed using Periodic acid–Schiff stain.

**Supplementary Fig 2.** Bioinformatics analysis of proliferative PT in db/db mice compared to db/m mice. **A** The tox list **B** canonical pathway of the DGEs of proliferative PT in db/db mice compared to db/m mice.

**Supplementary Fig 3.** The tox list of **A** module 1 and **B** module 6 of PT of WGCNA

**Supplementary Fig 4.** Bioinformatic analysis of MCD in db/m mice. **A** The KEGG analysis of module 1 (MCD of db/m mice) of WGCNA. **B** The network of module 1. **C** Violin plot of hub gene MGST1 expression in MCD of db/m and db/db mice.

**Supplementary Fig 5.** MC-secreted SEMA3C contributed to endothelial-mesenchymal transition (EndoMT) and increased permeability in GECs of DKD through NRP1 and NRP2 pathway. **A** The viability assay of GEC treated with different concentrations of SEMA3C (0 and 10 ng/ml) for 48 h. **B** After transfection with SEMA3C siRNA (20 nM) or normal control (NC) (20 nM) for 24 h, the efficiency of SEMA3C protein knockdown in the supernatant of MC treated with HG for 48 h was measured using ELISA. **C** After pretreatment with IgG (5 μg/ml) or NRP1 neutralizing antibody (nAb, 5 μg/ml) in GEC for 1 h, GEC was cultured with the supernatant of HG-treated MC for 48 h. E-cadherin, N-cadherin and vimentin expression in cultured GECs was examined using western blotting. **D** Permeability of cultured GECs was assessed using transendothelial permeability assay. **E** After pretreatment with IgG (20 μg/ml) or NRP2 nAb (20 μg/ml) in GEC for 1 h, GEC was cultured with the supernatant of HG-treated MC for 48 h. E-cadherin, N-cadherin and vimentin expression in GECs was assessed using Western blotting. **F** Permeability of cultured GECs was evaluated using transendothelial permeability assay. **G** After pretreatment with IgG (5 μg/ml) or NRP1 nAb (5 μg/ml) in GEC for 1 h, GEC was cultured with SEMA3C (10 µM) for 48 h. E-cadherin, N-cadherin and vimentin expression in GECs was assessed using Western blotting. **H** Permeability of cultured GECs was determined using transendothelial permeability assay. **I** After pretreatment with IgG (20 μg/ml) or NRP2 nAb (20 μg/ml) in GEC for 1 h, GEC was cultured with SEMA3C (10 µM) for 48 h. E-cadherin, N-cadherin and vimentin expression in GECs was assessed using Western blotting. **J** Permeability of cultured GECs was evaluated using transendothelial permeability assay.
