# Supplementary material for: Single-cell transcriptomic profiles in the pathophysiology within the microenvironment of early diabetic kidney disease
Source: Cell Death Dis. 2023 Jul 17;14(7):442. doi: 10.1038/s41419-023-05947-1 (PMC10352247; doi:10.1038/s41419-023-05947-1)
Supplement: Supplementary file 20 — Supplementary Table 18 [file 41419_2023_5947_MOESM20_ESM.docx]

Supplementary Table 18. The clinical characteristics of human participates

|  | Normal Individuals  N = 24 | Type 2 Diabetes  N = 48 | p-value |
| --- | --- | --- | --- |
| Age, years | 61.6 ± 8.9 | 63.0 ± 10.2 | 0.56 |
| Sex (male), % | 41.7 | 68.7 | 0.02 |
| Fasting blood glucose, mg/dL | 92.4 ± 12.7 | 136.6 ± 30.0 | <0.001 |
| Blood urea nitrogen, mg/dL | 13.8 ± 3.3 | 21.4 ± 9.4 | <0.001 |
| Creatinine, mg/dL | 0.8 ± 0.2 | 1.3 ± 0.8 | <0.001 |
| Urine albumin/creatinine ratio, mg/g | 4.0 (2.3,6.0) | 118.0 (10.4,854.2) | <0.001 |

Data are expressed as number (percentage) for categorical variables and median (25^th^, 75^th^ percentile) for continuous variables, as appropriate
