# Supplementary material for: Single-cell transcriptomic profiles in the pathophysiology within the microenvironment of early diabetic kidney disease
Source: Cell Death Dis. 2023 Jul 17;14(7):442. doi: 10.1038/s41419-023-05947-1 (PMC10352247; doi:10.1038/s41419-023-05947-1)
Supplement: Supplementary file 24 — Supplementary Table 22 [file 41419_2023_5947_MOESM24_ESM.docx]

Table 22. The biomarkers of different kidney cells

| **Kidney cell type** | **Surface biomarker** |
| --- | --- |
| Proximal tubule | AQP1, LRP2, SLC13A1 |
| Thick ascending limb | SLC12A1, UMOD |
| Distal convoluted tubule | SLC12A3, SLC8A1 |
| Connecting tubule-intercalated cell | SLC26A4, SCNN1A |
| Connecting tubule-principal cell | AQP2, FXYD4, STC1 |
| Medullary collecting duct | UPK1B, UPK3A |
| Mesangial cell | PDGFRB, CFH, ZEB2, COL12A1 |
| Podocyte | NPHS1, NPHS2 |
| Endothelial cell | PECAM1, CDH5 |
| B cell | CD19 |
| T cell | CD3E |
| NK cell | NCR1 |
| Monocyte | CSF1R, ADGRE1 |
| Granulocyte | CXCR2, CSF3R |
