# Supplementary material for: Single-cell transcriptomic profiles in the pathophysiology within the microenvironment of early diabetic kidney disease
Source: Cell Death Dis. 2023 Jul 17;14(7):442. doi: 10.1038/s41419-023-05947-1 (PMC10352247; doi:10.1038/s41419-023-05947-1)
Supplement: Supplementary file 25 — Supplementary Table 23 [file 41419_2023_5947_MOESM25_ESM.docx]

| **Codex IF** | Catalog number | Diluted ratio |
| --- | --- | --- |
| **Abcam** | | |
| Sema3c | ab135842 | 1:50 |
| Spp1 | ab283669 | 1:2000 |
| Aqp1 | ab178352 | 1:50 |
| Aqp4 | ab282586 | 1:200 |
| Scnn1a | ab272878 | 1:100 |
| Pdgfrb | ab69506 | 1:200 |
| **Proteintech** | | |
| Agt | 11992-1-AP | 1:200 |
| Slc12a1 | 18970-1-AP | 1:200 |
| GPX4 | 67763-1-Ig | 1:2000 |
| **Sigma** | | |
| Anti-Hu/Mu Ki67(B56)-BX047 | SKU 4250019 | 1:500 |

| **IHC** | Catalog number | Diluted ratio |
| --- | --- | --- |
| **Abcam** | | |
| GPX4 | ab125066 | 1:200 |
| **Invitrogen** | | |
| Aqp 1 | PA5-53954 | 1:100 |

| **Protein** | Catalog number | | Diluted ratio |
| --- | --- | --- | --- |
| **BD Biosciences** | | | |
| N-cadherin  E-cadherin  vimentin | 610921  610182  550513 | | 1:2000  1:2000  1:2000 |
| **EMD Millipore** | | | |
| GAPDH | mab374 | | 1:3000 |
| **cell signalling technology** | | | |
| Neuropilin-1  Neuropilin-2 | 3725  89189 | | 1:1000  1:1000 |
| **R&D Systems** | |  | |
| Neuropilin-1  Neuropilin-2 | AF3870  AF2215 | | 5 µg/mL  20 µg/mL |
|  | |  | |
| **ELISA Antigen** | | Catalog number | |
| **CusaBio** | | | |
| Ceruloplasmin | | Cat.CSB-E07023m-1 | |
| MyBioSource  Human SEMA3C  Mouse SEMA3C | | Cat.MBS7246650  Cat.MBS2533468 | |
| **R&D Systems** | | | |
| Mouse TIM-1/KIM-1/HAVCR | | Cat.MKM100 | |
| Mouse Lipocalin-2/NGAL | | Cat.MLCN20 | |
| Mouse/Rat Osteopontin (OPN) | | Cat.MOST00 | |
|  | |  | |
| **Magnetic Luminex Assay** | | Catalog number | |
| **R&D Systems** | | | |
| Human Osteopontin (OPN) | | Cat.LXSAHM-01 | |

| description | Mature microRNA sequence |
| --- | --- |
| ON-TARGETplus Human SEMA3C(10512) siRNA | Target Sequence:  CAAAUGAUGCUAAGGUGUA  GCAUUCAGCCACUCAGAAA  GCUCGAAUAUGUCCUAAUG  GCACGAAACAGAUUCAUUC |
| ON-TARGETplus Non-targeting | Target Sequence:  UGGUUUACAUGUCGACUAA  UGGUUUACAUGUUGUGUGA  UGGUUUACAUGUUUUCUGA  UGGUUUACAUGUUUUCCUA |
